# Application of Nanoparticles in Human Nutrition: A Review

**DOI:** 10.3390/nu16050636

**Published:** 2024-02-25

**Authors:** Ammar B. Altemimi, Halgord Ali M. Farag, Tablo H. Salih, Farhang H. Awlqadr, Alaa Jabbar Abd Al-Manhel, Italo Rennan Sousa Vieira, Carlos Adam Conte-Junior

**Affiliations:** 1 Department of Food Science, College of Agriculture, University of Basrah, Basrah 61004, Iraq; alaa.abd@uobasrah.edu.iq; 2 College of Medicine, University of Warith Al-Anbiyaa, Karbala 56001, Iraq; 3 Halabja Research Center, Halabja Technical College Applied Science, Sulaimani Polytechnic University, Sulaimani 46002, Iraq; halgord.farag@spu.edu.iq (H.A.M.F.); tablo.salih.tcas@spu.edu.iq (T.H.S.); farhang.hamid.a@spu.edu.iq (F.H.A.); 4 Harem Research Center, Department of Nutrition and Diet Therapy, Harem Hospital, Sulaimani 46001, Iraq; 5 Center for Food Analysis (NAL), Technological Development Support Laboratory (LADETEC), Federal University of Rio de Janeiro (UFRJ), Cidade Universitária, Rio de Janeiro 21941-598, RJ, Brazil; conte@iq.ufrj.br

**Keywords:** bioactive compounds, cancer, health human, nanotechnology, nutraceuticals, phytochemicals

## Abstract

Nanotechnology in human nutrition represents an innovative advance in increasing the bioavailability and efficiency of bioactive compounds. This work delves into the multifaceted dietary contributions of nanoparticles (NPs) and their utilization for improving nutrient absorption and ensuring food safety. NPs exhibit exceptional solubility, a significant surface-to-volume ratio, and diameters ranging from 1 to 100 nm, rendering them invaluable for applications such as tissue engineering and drug delivery, as well as elevating food quality. The encapsulation of vitamins, minerals, and antioxidants within NPs introduces an innovative approach to counteract nutritional instabilities and low solubility, promoting human health. Nanoencapsulation methods have included the production of nanocomposites, nanofibers, and nanoemulsions to benefit the delivery of bioactive food compounds. Nutrition-based nanotechnology and nanoceuticals are examined for their economic viability and potential to increase nutrient absorption. Although the advancement of nanotechnology in food demonstrates promising results, some limitations and concerns related to safety and regulation need to be widely discussed in future research. Thus, the potential of nanotechnology could open new paths for applications and significant advances in food, benefiting human nutrition.

## 1. Introduction

In the contemporary era, it is challenging to identify a field of human activity that does not leverage the capabilities of nanotechnology. Modern nanomaterials have attained sophisticated levels of complexity and have been applied in numerous scientific and industrial domains, including electronics, automotive engineering, construction, medicine, pharmaceuticals, environmental protection, and the food industry [1]. In this context, nanotechnology stands out as a revolutionary technology, driving progress in and having an enduring impact on various human endeavors, notably in the realms of food, medicine, and agriculture, with a particular emphasis on human nutrition [2].

Continuous innovation in nanosized product development necessitates novel technologies for emerging applications, especially in the food industry, paving the way to create safer and healthier food. Nanomaterials, functioning as sensors, are pivotal in ensuring food safety by detecting and identifying germs, viruses, and chemicals [1,3]. Furthermore, scientific evidence indicates that nanotechnology holds the potential to enhance the thermal stability, water solubility, and oral bioavailability of nutrients [4].

Nanoparticles (NPs) employed for delivering edible substances are nanometric materials ranging in size from 1 to 100 nm, created through various techniques. They possess notable chemical and physical attributes, such as their solubility, color, strength, infusibility, and high surface-to-volume ratio [5]. These characteristics make NPs valuable in applications such as tissue engineering, cell treatment, drug delivery, diagnostics, biomaterials, and signaling molecules. Furthermore, nanobiotechnology may facilitate the targeted delivery of drugs to specific organs in the human body by overcoming existing challenges [6,7].

Nanomaterials contribute not only to human health but also to the quality and safety of food. The inclusion of essential functional dietary components, such as vitamins, phytochemicals, minerals, and antioxidants, is crucial for achieving and maintaining optimal human health while mitigating the risk of various diseases [8]. The term “nutraceuticals,” a combination of “pharmaceutical” and “nutrition”, refers to substances obtained from food with physiological, preventative, or therapeutic benefits beyond basic nutritional needs [8]. Despite the inherent challenges of chemical instability, unfavorable taste, and limited solubility properties, NP technology, utilizing fewer excipients to enhance micronutrient solubility, has gained widespread acceptance in recent years [9].

To address challenges in the integration of bioactive substances into food products, NP-based delivery systems can be employed. Precise nanoscale delivery systems are crucial for encapsulating various micronutrients, considering their properties and nature. Techniques such as nanocomposite formation, nanofibers, and nanoemulsification are employed to encapsulate materials in small dimensions, thereby enhancing the efficiency of delivering bioactive chemicals [10].

**Figure 1:**
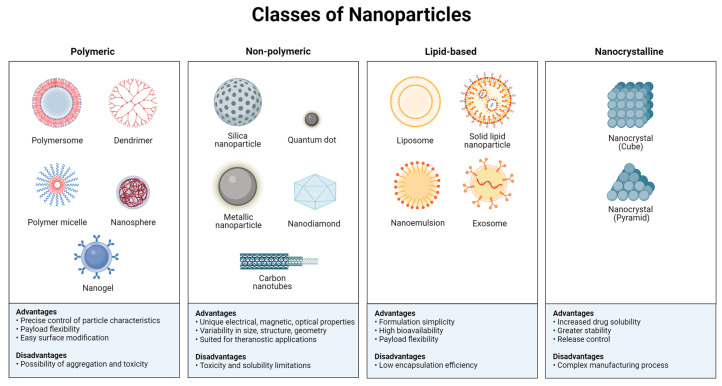
Main classes of nanoparticles (NPs), including polymeric, non-polymeric, lipid-based, and nanocrystalline materials for encapsulation of nutrients and bioactive compounds from foods.

Several delivery systems of NPs for the encapsulation of nutrients and bioactive compounds from foods have been proposed in recent years [2]. The main classes of NPs include (i) polymeric nanomaterials (polymersomes, polymeric micelles, dendrimers, nanospheres, and nanogels); (ii) non-polymeric nanomaterials (silica NPs, quantum dots, nanodiamonds, carbon nanotubes, and metallic NPs); (iii) lipid-based nanomaterials (liposomes, solid lipid NPs, nanoemulsions, and exosomes); and (iv) nanocrystalline materials (cubic and pyramidal nanocrystals) (Figure 1) [11,12,13].

Nutrition-based nanotechnology and nanoceuticals represent designations for several commercially available dietary supplements. Explorations into vitamin spray-dispersed nanodroplets reveal a potential strategy to enhance the absorption of nutrients like iron, curcumin, and folic acid. Nano-sized powders, particularly nanocochleate structures, contribute to improved nutrient absorption [10]. These methods effectively deliver nutrients to cells without impacting the taste or color of the food.

The primary method of supplement manufacturing involves encapsulating probiotics and other goods in zinc (Zn) and iron (Fe) nanostructured capsules. Food supplement NPs, due to their size, exhibit a heightened activity compared to that of other supplements as they interact more effectively with human cells. Nanocapsules enhance the bioavailability of antioxidants, essential oils, coenzyme Q10, vitamins, phytochemicals, and minerals. Simultaneously, NP technology has garnered recognition for its ability to utilize minimal additives to improve the solubility of micronutrients [14]. The encapsulation of bioactive components in NP-based delivery systems addresses these challenges, but it requires accurate and suitable nanoscale delivery systems tailored to the specific qualities and characteristics of the micronutrients. Thus, this review aims to underscore the significance of nanotechnology in enhancing the bioavailability and absorption of both macronutrients and micronutrients using customized delivery systems.

## 2. The Role of Nanotechnology in Human Nutrition

Nanotechnology plays a significant role in human nutrition, offering countless opportunities to improve food quality and safety [15]. In recent years, food scientists have dedicated themselves to developing new food products with enhanced functional properties [16]. For example, producing nanocolloids for food applications has emerged as an alternative for encapsulating nutrients and bioactive compounds, improving their absorption and bioavailability in the gastrointestinal system [17]. These nanostructures are typically produced from proteins, polysaccharides, or lipid compounds, which may consist of a single molecular species or a combination of several molecular components [18].

Commonly observed nanocolloids include nanoemulsions, nanomicelles, nanocapsules, or similar structures [2]. The demand for nanocapsules is growing due to their application in smart medicine delivery within the body and the creation of liposomal nanocapsules used in food science, medicine, and agriculture [19]. Nanocapsules offer protection against degradation during processing, enabling the potential for the controlled and/or sustained release of active components [20]. Furthermore, these nanomaterials can also include therapeutic substances, combining a core with one or more active ingredients and a protective matrix shell, with sizes ranging from 10 to 1000 nm [20].

Examples of substances used to produce nanocapsules include polymeric NPs made from nonionic surfactants, phospholipids, macromolecules, and either aqueous or oil cores. Lately, there has been an increasing interest in creating lipid-based nanocarriers (LNCs), especially with an organogel structure [21]. This structure consists of a core composed of a combination of liquid and solid lipids enveloped by a polymer wall. The stability of these nanocarriers is ensured by the presence of surfactants, such as polyethylene glycol sorbitan monolaurate (Tween^®^ 20) micelles [18,22].

The manufacturing of nanocarriers employs diverse techniques, including monomer polymerization in a water-based solution, interfacial polymerization on the surface of an existing polymer, emulsion polymerization, and the arc discharge process [23]. Additional examples include liposomal nanocapsules and spherical bilayer vesicles created through the dispersion of polar lipids in hydrophilic fluids [24]. There is significant interest in protein- and NP-based nanocapsules across all disciplines [25]. Proteins possess distinctive functional characteristics, such as their interaction with water and their ability to form gels or emulsions, making them excellent for enclosing bioactive chemicals [26]. Proteins and other nutraceuticals, like bioactive peptides, antioxidants, vitamins, and probiotics, are considered vital micronutrients to our diet [27]. Their effectiveness in preventing diseases relies on the continued presence of active components in the body post-ingestion [19].

Delivery techniques using synthetic chemicals in biomedicine or pharmaceuticals may not always be applicable in the food industry due to the prevalent need for safe compounds. Therefore, the nanoencapsulation of probiotics emerges as a safe alternative for the delivery of bioactive food components [28]. Probiotics, described as living combinations of bacterial species with beneficial effects upon ingestion, offer health advantages like enhanced general well-being, reduced blood cholesterol levels, and improvements in the body’s immune system [29].

In recent years, the nanoencapsulation of probiotics has gained prominence from producing nanofibers using electrospinning. Encapsulating probiotic strains in nanofiber mats made of corn starch and sodium alginate has significantly enhanced their durability and viability, surpassing that of non-encapsulated cells [30]. Utilizing electrospinning to produce nanofiber mats composed of sodium alginate and starch has demonstrated superior protective properties compared to microencapsulation or nanoencapsulation using a single biopolymer [31]. These nanofiber mats have been incorporated into dairy products such as yogurt, milk, cheese, puddings, and drinks [32].

Although the use of nanoencapsulated probiotics in foods offers several advantages in protecting the probiotics and greater efficacy in delivery to the intestine, there are some disadvantages, such as high costs, complexity in terms of production, concerns about adverse nanoscale effects, complex regulations, sustainability, and potential instability during processing. These aspects must be considered during the development and production of these products [33].

## 3. Nanoencapsulation of Bioactive Nutraceuticals 

**Figure 2:**
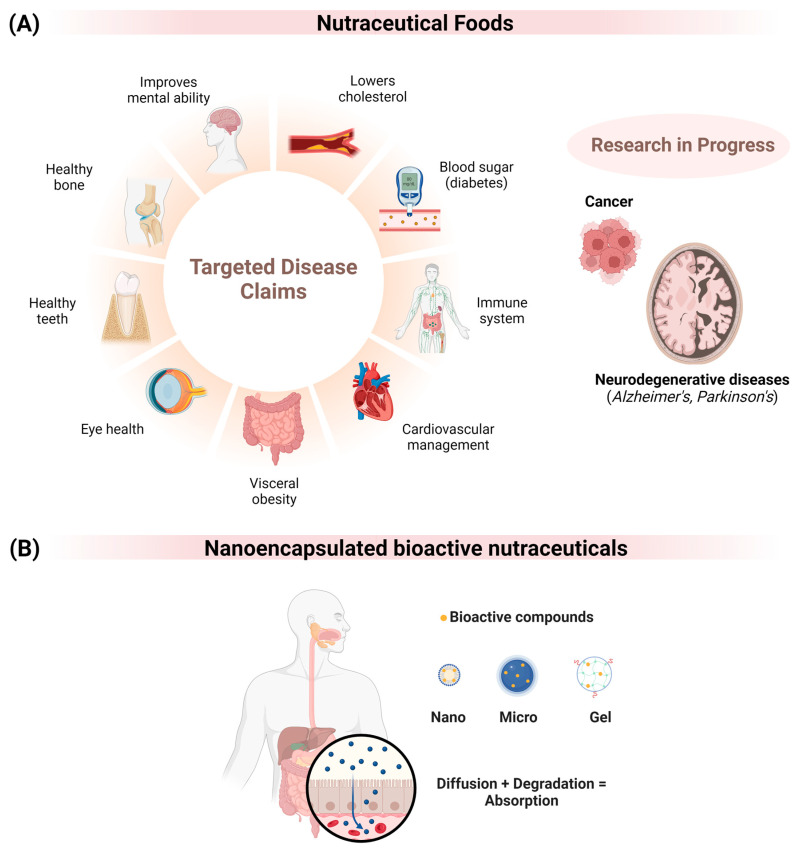
(**A**) Nutraceutical bioactive compounds can prevent and treat various diseases. Recent research has focused on treating cancer and neurodegenerative diseases, including Alzheimer’s and Parkinson’s. (**B**) Nanoencapsulated foods can be an alternative to improve the stability, absorption, and bioavailability of bioactive nutraceuticals in relation to microencapsulated analogues.

There has been a significant increase in our understanding of and applications of strategies aimed at preserving health, enhancing well-being, and addressing common or chronic health conditions through dietary choices. Nutraceutical foods have been extensively researched for their potential in preventing and treating various disorders (Figure 2A) [34]. However, these bioactive compounds face several limitations, including insufficient stability, limited water solubility, and restricted bioavailability [35]. Consequently, encapsulating these compounds in a suitable carrier is advantageous as it improves their water solubility and protects them from damage caused by environmental and biological factors [36].

Microencapsulation can enhance the stability and bioactivity of bioactive nutraceuticals. Encapsulated particles are typically categorized into microcapsules (0.2–5000 µm), macrocapsules (larger than 5000 µm), and nanocapsules (smaller than 0.2 µm) [37]. Various naturally occurring nanoencapsules can be found, such as casein micelles in milk (measuring less than 100 nm) [38], mitochondria (ranging from 500 to 10,000 nm) [39], and viruses (measuring between 10 and 300 nm) [40]. Nonetheless, large particles and a high concentration of wall components can pose certain limitations, impacting their absorption in the gastrointestinal tract (GIT) (Figure 2B) [41].

Due to their larger size and smaller specific surface area, macro- and microparticles may present a poor absorption of nutrients and other encapsulated compounds in the GIT [42]. Larger particles have a prolonged dissolution time, which can delay the body’s release and absorption of nutrients before passing through the digestive system. These materials can also clump together, resulting in an uneven distribution throughout the GIT and affecting absorption in certain areas [43]. The high concentration of wall components in encapsulated particles can interfere with the degradability of the particles in the GIT. This interference can affect the release of encapsulated nutrients, making them less available for absorption. Furthermore, some wall materials used in encapsulation may confer resistance to digestive enzymes in the GIT [44]. This resistance can result in a slower release of the encapsulated nutrients, affecting their absorption.

The nanoencapsulation of bioactive components commonly provides notable benefits [45]. The bioavailability of food ingredients significantly increases when the surface area available for absorption expands [46]. This is particularly crucial for flavoring substances with limited ability to dissolve in a liquid and/or for flavors and odors detectable at very low levels [47]. Some bioactive compounds have limited absorption and undergo rapid metabolism after ingestion, hindering their ability to reach target organs and produce beneficial effects [48]. Nanocarriers significantly enhance nutrient absorption by safeguarding them during digestion and facilitating controlled release throughout the digestive process [49].

Additionally, the nanoencapsulation of bioactive compounds minimizes unwanted interactions with other food components and protects against spoilage during and after consumption [47]. The solubility of ingredients with low water solubility, such as omega-3 fish oil, can be improved by forming a micelle network or preventing interactions with other substances in the matrix [50]. This prevents discoloration, reduces off-flavors by masking tastes and smells, releases mineral components in a controlled manner, ensures optimal preservation during production and storage, and enhances the physical properties of the product [51]. Nanoencapsulated bioactive compounds can release their content in a regulated and prolonged manner, thereby enhancing their solubility in water and bioavailability for biological processes [52]. The improved water solubility and controlled release of food components when encapsulated at the nanoscale can be attributed to specific factors [45]. Table 1 displays a selection of bioactive compound release systems presently accessible worldwide [53].

Lately, there has been a significant emphasis on developing effective methods for the nanoencapsulation of minerals in human nutrition [54,55]. This endeavor seeks to improve the creation of innovative functional food formulations and the fortification process of food [52]. Most studies have concentrated on improving the mineral composition of dairy products and table salt by utilizing nanoencapsulated minerals, particularly iron, calcium, zinc, and iodine [56]. The primary materials used to enhance dairy products consist of electrolytic iron, iron sulfate, iron sulfate heptahydrate, iron(II) glycine sulfate, ammonium iron(II) sulfate, and iron(III) citrate, all of which are iron compounds in encapsulated form [57]. Tricalcium phosphate and calcium citrate, two calcium forms enclosed in a protective covering, have been utilized to enhance the nutritional content of soy yogurt and soy milk [58]. The typical formulations to incorporate iodine and iron into edible salts are potassium iodide and potassium iodate for iodine, iron(II) fumarate, and iron(II) sulfate for iron [59]. Attempts have been made to produce fortified bread products by incorporating minerals into flours through nanoencapsulation, which act as efficient carriers [56]. Table 2 displays various products in which minerals are encapsulated at the nanoscale.

## 4. Bioavailability of Nanoencapsulated Bioactive Compounds

Lately, there has been a focused research effort aimed at developing innovative nanocarriers with the potential for use in producing functional foods [54]. However, only a limited number of these products have made it to the market [49]. These systems have been commonly produced using inorganic (iron, silver, calcium, magnesium, selenium, and titanium oxide) and organic nanomaterials (lipids, proteins, and polysaccharides) [64]. The prevalence of in vitro testing in a controlled laboratory setting outweighs in vivo testing, necessitating additional studies in living organisms to establish the effectiveness and safety of these NPs/capsules [65].

The “rule of five” dictates that 90% of oral medications in the pharmaceutical industry must meet at least three out of four specific criteria to ensure favorable oral bioavailability [66]. These criteria include the following: (i) a molecular weight (MW) less than or equal to 500 Da; (ii) the logarithm of the partition coefficient (log(P)) is less than or equal to five; (iii) no more than 10 hydrogen bond acceptors (HBAs); (iv) no more than five hydrogen bond donors (HBDs); and (v) some writers also include a requirement for a polar surface area (PSA) of no more than 140 Å^2^ and a maximum of 10 rotatable bonds (NRotB) [67]. These guidelines are based on the findings related to oral bioavailability. The uptake of bioactive nanocapsules is influenced by the properties of the bioactive compounds, the specific nanocapsules used, and the ambient conditions in which they are present [68].

Bioactive substances can be released from nanocapsules through various processes, including diffusion, dissolution, erosion, swelling, osmosis, degradation, and fragmentation [45]. For example, diffusion involves the movement of a bioactive substance from the interior to the exterior of an encapsulation system [69]. This movement is influenced by the substance’s solubility within the contained system and its ability to permeate through the material of the capsule [70]. Several factors affect the release rate of bioactive compounds, including the molecular weight, polarity, and vapor pressure of the bioactive molecule itself and the polarity, physical state, interactions, and rheology of the encapsulating system [45].

**Figure 3:**
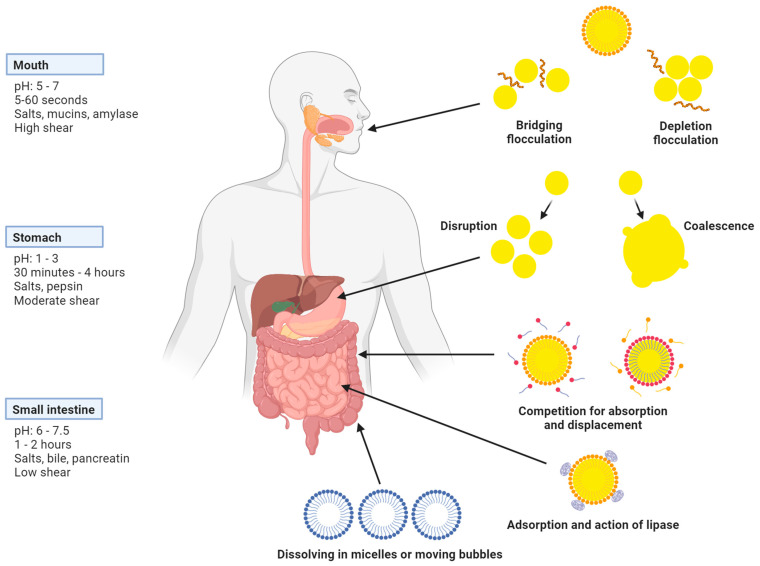
Possible nanoemulsion structural changes during gastrointestinal transit.

In recent years, lipid nanoemulsions have been a trend in the food industry. Nanoemulsions have demonstrated significant efficacy in enhancing the absorption, effectiveness, breakdown, durability, safety, excellence, and sensory characteristics of nutritional elements and organic extracts, particularly lipophilic bioactive compounds like vitamins and nutraceuticals [71,72]. Before being incorporated into food fortification, these substances are emulsified. Therefore, it is essential to comprehend the fate of bioactive-fortified nanoemulsions in the GIT [73,74]. Figure 3 shows some physicochemical and biochemical processes involved in lipid digestion in the GIT.

According to Figure 3, the size and interfacial characteristics of oil droplets can play a key role in the digestibility of lipid droplets and the release of bioactive components in the GIT [75]. Salts in the mouth can influence nanoemulsion stability and ionic strength. Saliva mucin either bridges or depletes flocculation, causing the aggregation of nanodroplets. Oil droplets may aggregate in the stomach due to its low pH, high ionic strength, and shear conditions. Gastric lipase initiates the hydrolysis of lipid droplets, with pancreatic lipase predominantly hydrolyzing them in the small intestine. Bile salts in the gastrointestinal fluids displace emulsifier molecules from the oil–water interface, promoting enzyme interaction [76]. During lipolysis, free fatty acids and monoacylglycerols accumulate on the oil’s surface. Due to their elevated surface activity, long-chain free fatty acids (FAs) and monoglycerides (MGs) can inhibit lipolysis by displacing lipase enzymes. Bile salts dissolve digestion products in mixed micelles on the oil droplet’s surface, facilitating complete digestion. Calcium ions can form insoluble soaps with long-chain free fatty acids, effectively removing them from droplet surfaces [77]. Ultimately, the breakdown products of fat and fat-soluble compounds are dissolved in micelles or single-layered structures made of phospholipids, which attach to the inner lining of the intestines [78]. Understanding these gastrointestinal processes is crucial for formulating nanoemulsions that enhance the bioaccessibility and bioavailability of bioactive substances [79].

Table 3 summarizes recent studies exploring the potential advantages of incorporating active lipophilic substances into nanoemulsions [80]. It is evident that combining hydrophobic bioactive chemicals with a lipid phase aids in their absorption post-digestion. Studies indicate a positive correlation between nanoemulsions’ droplet size and encapsulated bioactive chemicals’ bioaccessibility. This correlation is linked to the enhanced digestibility of the lipid phase [81]. For example, the bioaccessibility of β-carotene was 60% after the in vitro digestion of nanoemulsions, but only 34% after the digestion of traditional emulsions [82]. The reduced bioaccessibility of carotenoids in emulsions with larger droplets can be attributed to a greater quantity of undigested oil retaining β-carotene and a lesser quantity of mixed micelles available to dissolve β-carotene. Decreasing the droplet size often enhances the bioavailability of hydrophobic bioactive compounds [82].

Finally, nanoemulsions manufactured from digestible lipids, such as triglycerides, are expected to undergo rapid digestion within the GIT. However, their behavior may vary when composed of indigestible lipids, such as mineral or taste oils [83,84]. In this scenario, epithelial cells in the GIT have the ability to absorb tiny lipid droplets. This effect was observed in the study by Yu and Huang (2013) [85], which evaluated the cytotoxicity of nanoemulsions based on whey protein isolate against Caco-2 cell monolayers and HepG2 cells. The researchers observed no harmful effects of nanoemulsions with droplet diameters less than 200 nm on Caco-2 cells found in the small intestine. However, compared to traditional emulsions, a more pronounced decrease in HepG2 cell viability (liver cells) was observed following exposure to nanoemulsions. These findings suggest that while nanoemulsions can improve the absorption of specific food components, further research is needed to understand their impact on the body and potential risks. Future research employing cell culture and animal models is recommended to monitor the possible gastrointestinal fate of both digestible and nondigestible nanoemulsions [85]. Table 4 presents some nanoemulsion-based products available on the global market.

**Table 3:** Recent studies that have explored the potential advantages of incorporating bioactive compounds into nanoemulsions.

Complex	Classifications	Operational Capabilities	References
Antioxidants	Flavonoids, tocopherols, and polyphenols are compounds with antioxidant properties.	Preventing coronary heart disease, cancer, and urinary tract disease.	[86]
Carotenoids	The compounds β-Carotene, lycopene, lutein, and zeaxanthin.	Prophylaxis against coronary heart disease, macular degeneration, cataracts, and cancer.	[87]
Essential oils (EOs)	The herbs oregano, sage, clove, and mint, as well as the compound limonene, among others.	Effect of flavorings on microbiological growth.	[88]
Fatty acids (FAs)	Omega-3 fatty acids, conjugated linoleic acid, and butyric acid.	The benefits of this intervention include reducing the risk of stroke, cancer, immune response problems, and heart disease. It also enhances optical acuity, bone health, and emotional well-being.	[89]
Phytosterols	Stigmasterol, β-sitosterol, and campesterol.	Coronary heart disease prevention.	[90]
Quinones	Coenzyme Q10	Antioxidant activity	[91]

**Table 4:** Products on the market that are made up of or contain active nanoemulsions [92].

The Company	The Item	Features of the Product
Assam Company India Ltd.	Gold nano-tea	Gold nano-tea stimulates the immune system and prevents sickness, making it a wonderful daily supplement for cancer patients.
LivOn Labs	Lypo-spheric vitamin C	Smart liposomal Nano-spheres^®^ with Lypo-Spheric™ Vitamin C enters the bloodstream through the intestinal wall. They cross a narrow nutrient transport channel that limits non-liposome-encapsulated vitamin C absorption.
NutraLease Ltd. Israel	NSSL food and beverage supplements	Patent-pending NSSL technology is involved. Nutraceuticals and medications are transported via nano-sized vehicles (micelles with a diameter of 30 nm).
RBC Life Sciences^®^ industrial company	Nanoceuticals™ Slim Shake Chocolate	Nanoscale components scavenge more free radicals, boost hydration, balance pH, reduce lactic acid during exercise, lower food surface tension, and increase moisture and nutrient absorption.
Shemen Industries Ltd.	Canola active oil	Compressed nanodrops produce NSSL. These micelles transport water- and lipid-insoluble vitamins, minerals, and phytochemicals. During digestion, food micelles remain intact. Large bile acid micelles compete with cholesterol for phytosterols from tiny micelles. Micelle phytosterols block intestinal cholesterol absorption. This unique approach helped Shemen Industries make canola active oil.
Shenzhen (later known as China Industry & Trade Co., Ltd).	Nano-tea	Gold Nano-tea’s anti-aging qualities revitalize the body and keep the skin young.
PureHealth^®^ Research	The product is nano-powered omega-3 with DHA and EPA, a non-GMO fish oil supplement that comes in a package of 60 soft gels.	Employs nanotechnology to turn large molecules into millions of little ones for the body to absorb like a sponge. It can benefit the heart, brain, joints, and skin.
Tip Top, India	Tip Top UP^®^ DHA, an omega-3 fatty acid	The bread is enhanced with nanocapsules that contain omega-3 DHA-rich tuna fish oil.

Legend: DHA = docosahexaenoic acid; EPA = eicosapentaenoic acid; GMO = genetically modified organism; NSSL = self-assembled structured liquid.

## 5. Application of Nanoparticle-Based Nanosensors in Food

Nanosensors are bioanalytical instruments created by combining biological receptors and nanomaterials, significantly impacting food safety [93]. Their unique properties, such as optical and electrical enhancements and their high surface-to-volume ratio, directly influence their efficacy [94]. These devices offer numerous advantages, including swift pathogen detection and the cost-effective production of crucial bioproducts for analysis compared to larger alternatives [95]. Moreover, they excel in identifying toxins, chemicals, pesticides, and agricultural contaminants [96].

Using analytical techniques like a flow analysis or physicochemical methods such as surface plasmon resonance, voltammetry (pulsed, cyclic), amperometry, and bioluminescence, nanosensors have diverse applications [97]. They are indispensable for monitoring food product freshness and evaluating packaging integrity [98]. Nanosensors can be manufactured from various nanomaterials, such as metallic and non-metallic NPs, metal oxides, nanowires, carbon nanotubes (CNTs), nanorods, semiconductor NPs, nanofibers, or quantum dots, with active molecules [99].

Table 5 shows some potential applications of nanosensors in the food sector, spanning agricultural production, food processing, food safety, the packaging industry, and human nutrition. For example, pesticide residues were recently detected in food samples from a silver nanoparticle (AgNP) nanosensor using the surface-enhanced Raman scattering (SERS) method [100]. Spherical and highly monodisperse AgNPs created remarkable electromagnetic fields during SERS activities to measure phosmet in a methanol–water solution and Oolong tea. The results indicated that SERS coupled with AuNPs is a fast, sensitive, and reliable method for detecting and characterizing pesticide contaminants in food.

A nanosensor designed to detect oxygen levels in modified food packaging used activated UV radiation indicator dyes, relying on titanium (Ti) or tin (Sn) NPs and a redox-active dye such as methylene blue [101]. The food packaging was flooded with a gas (e.g., carbon dioxide or nitrogen). Subsequently, the dye was introduced into the packaging while the other was positioned externally. Both dyes used in these experiments showed a blue hue for the efficient detection of gases. In the study by Li et al. (2019) [102], highly sensitive nanosensors based on Pd NPs and reduced graphene oxide (rGO) modified with α-Fe_2_O_3_ were produced for ethylene detection in food packaging. The results revealed that the nanosensor could detect ethylene at low concentrations (10 ppb), which was the best among all of the ethylene gas sensors. The authors suggested that the high specific surface area of the flower-like porous structure, the catalysis of Pd NPs, and the chemically active defect sites of rGO contributed to the increased detection performance of the ethylene nanosensor. Thus, the nanomaterial showed potential for developing high-performance sensors in the food sector.

**Table 5:** Nanomaterials employed as nanosensors.

Agrifood Sector	Possible Uses	Occurrence of NPs	References
Agriculture	Nanosensor for the quantification of pesticides, such as phosmet.	Use of AgNPs and spectroscopic approach	[100]
Processing food	NPs for augmenting viscosity.	Starch NPs	[103]
Food packaging	Electromechanical nanosensors for ethylene.	The nanocomposite consists of palladium (Pd), rGO, and α-Fe_2_O_3_	[102]
Transportation of food	Aptasensors to detect microbial cells like *Staphylococcus aureus*.	PMB-capped silver NPs	[104]
Food safety	Food pathogen detection.	AuNPs functionalized with oligonucleotides	[105]
Human nutrition	Utilization of nanocapsules in food for targeted nutrient delivery.	ENVs	[106]
Key food ingredient instability detection	Folate and vitamin B9, ascorbic acid, glucose, fructose, sucrose, glutamic acid, caffeic acid, gallic acid catechol, and chlorogenic acid.	AgNPs, multi-walled carbon nanotubes, Ag, and tin dioxide NPs.	[107,108,109,110]

Legend: AgNPs = silver nanoparticles; AuNPs = gold nanoparticles; ENVs = edible nanoencapsulation vehicles; NPs = nanoparticles; rGO = reduced graphene oxide.

Nanosensors have also been widely applied for the detection of food pathogens. An electrochemical genosensor based on cadmium sulfide nanoparticles (CdSNPs) was developed to detect *Escherichia coli* O157:H7 in fresh meat samples to improve quality control and food safety [104]. The results showed the biosensor’s high sensitivity and selectivity for detecting *E. coli* O157:H7. Furthermore, the genosensor efficiently saw pathogenic bacteria in food and was superior to other electrochemical biosensors. In another study, a nanobiosensor based on gold nanoparticles (AuNPs) functionalized with oligonucleotides was applied to detect the SARS-CoV-2 virus on plant food surfaces [105]. The nanobiosensor used the loop-mediated isothermal amplification (LAMP) technique for the accurate detection (100% accuracy) of the SARS-CoV-2 virus, which was better than the standard quantitative reverse-transcription polymerase chain reaction technique (RT-qPCR). The nanobiosensor was proven to be sensitive, selective, and simple, providing a viable alternative for rapidly detecting SARS-CoV-2 in ready-to-eat vegetables.

**Figure 4:**
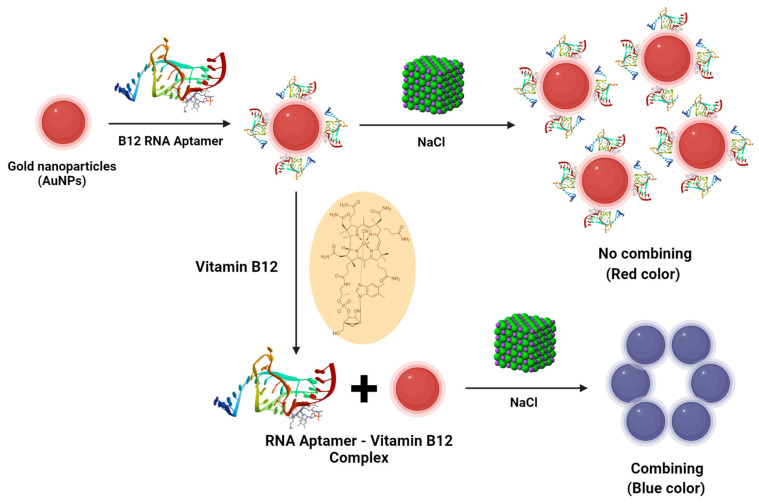
Vitamin-B12-induced structural variation identification by RNA aptamer and colorimetry.

Furthermore, NP-based nanosensors have been used in several human nutrition applications. For example, in the identification of ochratoxin A, a food microtoxin causing allergies, grape juice samples can be analyzed using a fluorescence assay for magnetic NPs and gold nanoparticles (AuNPs) [111]. On the other hand, quantum dots can serve as carriers for oral administration in immunotherapy for allergies to various foods [112]. Notably, a study has successfully developed a reliable and straightforward colorimetric sensor utilizing RNA aptamers and AuNPs to precisely measure vitamin B12, a crucial biomolecule found in foods and medications (Figure 4) [113].

## 6. Limitations and Further Research

The use of NPs in human nutrition holds significant promise, especially for antimicrobial applications, nutrient nanoencapsulation, and active food packaging [114]. Furthermore, nanomaterials have been widely explored for biomedical applications, including medical diagnoses, drug delivery systems, and disease treatments [115]. However, there are significant challenges and concerns related to the safety and potential risks of nanomaterials on human health and the environment [116]. These concerns are linked to consumer acceptance regarding the implementation of nanotechnology approaches in food [117].

One of the main limitations of using NPs in food is their toxicological potential [118]. Due to their small size, NPs can be introduced into the human body in various ways, including inhalation, ingestion, and absorption [98]. Their entry into cells and tissues can lead to adverse effects, such as inflammation, oxidative stress, and DNA damage [119]. Furthermore, the increased bioavailability of NPs can promote their accumulation in tissues and organs, potentially causing long-term damage [120]. NPs can also interact with various biological barriers in the human body, such as the blood–brain and placental barriers, which can have unforeseen consequences [121]. These interactions can interfere with the normal function of these barriers and lead to the entry of harmful substances into the brain or developing fetus [122].

NPs in contact with food can also lead to the risk of immunogenicity [123]. This means the immune system can recognize NPs as foreign bodies and trigger immune responses, leading to chronic inflammation or allergic reactions [124]. This can be especially problematic in long-term applications, where continuous NP exposure can lead to immunological complications [123]. Additionally, some NPs can persist in the environment or the human body for long periods, increasing the risk of long-term exposure and adverse effects [120]. Therefore, the ability of some NPs to accumulate in specific organs may further increase the risk of localized toxicity [125]. Finally, the unique properties of NPs can interfere with normal biological processes in the human body. For example, specific NPs can disrupt cellular communication, alter gene expression, or interfere with metabolic pathways, leading to unpredictable health consequences [126].

The regulation of NP-containing products is still under development and can vary significantly between countries and regions. Furthermore, the lack of standardization in the synthesis and characterization of NPs can make it difficult to assess their safety and efficacy [127]. Currently, the Food and Drug Administration (FDA) and the European Food Safety Authority (EFSA) of the European Union (EU) are the main international authorities with regulations and guidelines for using nanomaterials in food [98]. The FDA takes a risk-based approach to evaluate the safety of these materials, using the information provided by companies, including data on their toxicity, bioavailability, and potential adverse effects on human health [128]. The EFSA examines the scientific data available on nanomaterials in food, including toxicological, exposure, and behavioral studies of these materials in the human body. The EFSA provides guidance and recommendations to ensure consumer safety based on these risk assessments [127].

The potential risk level of NPs mixed into a food can vary due to their organic and inorganic nature. Inorganic or non-polymeric nanomaterials, including metals and metal oxides, and corresponding carbon-based nanomaterials, such as CNTs, GO, rGO, and nanodiamonds, have been identified as the groups of nanomaterials with the highest incidence of toxicity based on evidence from in vitro and in vivo studies [129]. On the other hand, organic nanomaterials derived from carbohydrates, lipids, and proteins, such as NPs, nanoemulsions, and nanocapsules, may present some toxicity, considering the ingredients and methods for their manufacture [130].

Recently, Kyriakidou et al. (2020) [131] evaluated the cytotoxicity of multi-walled carbon nanotubes (MWCNTs) against human epithelial adenocarcinoma cells (A549) as a potential material for the application of composites and food coatings. The authors observed that functionalized MWCNTs induced greater time- and dose-dependent toxic effects. Their SEM microscopy observations confirmed damage to the cell membrane and their cytotoxicity potential. Although some nanomaterials present toxicity at the cellular level, some mechanisms are still not wholly understood, primarily when imaging techniques evaluate cell interactions with the nanomaterial. In another study, GO and chitosan composite coatings prepared for biomedical and food packaging applications showed cytotoxicity against L929 fibroblast cells at high concentrations (above 60 ppm) and with increasing exposure times of 24 and 72 h [132]. That study suggested that the increased cellular toxicity at high concentrations was attributed to the induction of reactive oxygen species (ROS) by high concentrations of GO. These insights underscore the need for further research to elucidate the complex interplay between nanomaterials and biological systems, ensuring their safe and effective utilization in various food applications.

Edible nanoemulsions based on vegetable oils and a mixture of surfactants demonstrated toxicity against Caco-2 human intestinal cells that depend on particle size, concentration, and cell exposure time [133]. It has been suggested that the more significant level of cytotoxicity may result from the synergistic interaction of the formulation components, such as ethanol and the non-ionic surfactant ethoxylated sorbitan monooleate. Furthermore, incorporating a β-carotene-based compound considerably increased the toxicity of the nanoemulsion. In the study by Kunjiappan et al. (2020) [134], it was observed that high concentrations (200–500 μg × mL^−1^) of capsaicin-loaded lipid NPs had a more pronounced effect on the cytotoxicity of human embryonic lung fibroblast cells (WI-38). Although the results suggest some toxicity against WI-38 cells, applying lipid NPs in low concentrations may be safe as drug and nutrient delivery vehicles. Thus, it is essential to evaluate the cytotoxicity potential of these materials before their use in food matrices. This step is essential to provide accurate information to companies and consumers about the advantages and potential risks associated with NPs in food [135].

Nanoencapsulation is a dynamic and constantly evolving field, and although it has shown significant promise, there are still many gaps to be filled. Thus, exploring the different encapsulation materials available, their properties, and how they interact with other bioactive substances is essential. This can help optimize delivery efficacy by increasing the stability and bioavailability of encapsulated substances. Furthermore, more research is needed to better understand the mechanisms of the controlled release of encapsulated substances in the body. This includes investigating how factors such as pH, temperature, and environmental composition affect the release of encapsulated substances and developing more precise methods of controlling release.

Another important aspect is exploring the specific applications of nanoencapsulation in different fields, such as medicine, nutrition, cosmetics, and agriculture. Each area has challenges and opportunities that can benefit from nanoencapsulation technology in different ways. Furthermore, it is essential to carry out further safety studies to ensure that nanoencapsulation systems are safe for human and environmental use. With this in mind, epidemiological and toxicological studies on exposure to these nanomaterials require further investigation using in vivo approaches. Therefore, evaluating the possible adverse effects of encapsulation materials and encapsulated substances can help us better understand their fate in the body and the environment. Further research could reveal the potential of this technology, paving the way for new applications and significant advances in several areas from health to agriculture.

## 7. Conclusions and Outlook

The initial findings of our research indicate the potential advantages of utilizing nanoparticles (NPs) as carriers for active food components, such as antimicrobials or bioactive chemicals. Generally, smaller NPs exhibit a higher capacity to interact with microbial cells, leading to enhanced antimicrobial effects. Moreover, NPs containing bioactive compounds are more readily digested and absorbed, increasing their bioavailability. However, the optimistic outlook derived from recent data is based on a limited number of research publications with conclusive findings that merit further scrutiny and validation. Additional research must encompass diverse bioactive substances or other food ingredients encapsulated within NPs. This broader exploration will contribute to a more comprehensive understanding of the behavior and functionality of various types of lipid NPs.

Despite this, most research investigating lipid NPs activity is conducted using simplified models. Therefore, it is crucial to elucidate potential interactions between lipid NPs and other macromolecules present in food formulations, including proteins, carbohydrates, and fiber. This clarification is essential to provide accurate information to companies and consumers about the advantages of NPs in food. A pivotal step in assessing the benefits of incorporating NPs into food is to conduct tests integrating them into real food matrices. This assay aims to evaluate their functionality and biological fate. Furthermore, while lipid NPs often contain edible components and demonstrate a comparable digestibility pattern to traditionally emulsified lipids, their toxicological safety cannot be guaranteed.

Therefore, it is imperative to elucidate the gastrointestinal fate of lipid NPs upon entering the human stomach, assessing their biological outcome, tissue distribution, and potential bioaccumulation. Achieving this objective requires additional in vivo research alongside established in vitro approaches to compare and validate existing data.

## Figures and Tables

**Table 1:** Illustrations of functional/nutraceutical foods, including nanoencapsulated bioactive components [53].

Name of the Product	Bioactive Compounds	Claims Related to Health	Manufacturer’s Country of Origin
Creatine Nano by Azgard Nutrition.	Pure creatine nano monohydrate.	Increases muscle power.	Austria
IÖGO^®^	Source of calcium and vitamin D.	Helps in growing bones	Canada
B-B-Red Label Tea	6-Gingerol, withaferin A, glycyrrhetinic acid, and ursolic acid.	Enhancement of vitality (increase in natural killer cell activity).	Hindustan Unilever
Nano-Deca by Swayamacare	L-carnitine, L-arginine, ginko biloba (antioxidant), zinc, co-enzyme Q10.	Extends the length of time that the penis remains erect and enhances erectile function.	India
NANO™ COMBI: 7XORIGINAL 4XVEGGI HOT MEALS	Macro- and micronutrients, vitamins, and minerals.	Promotion of health	Netherland
NUTRASORB™ PROCES Ingredient	Polyphenols, gingerols, and beneficial alkaloids.	Promotion of health	New Jersey
NanOmega^®^	Vitamins (B6, B12, E, D3, and folic acid).	Important to eye, brain, and heart health.	USA
NanoMineral by NanoGreens	Contain 12 minerals in each packet.	Regulates blood glucose levels/improves metabolic function, assists in weight reduction, and improves muscle performance.	USA
NutriNoche Magnesium Supplement 30 PPM Nano Magnesium	Vitamins and minerals.	Crucial for the optimal operation of the kidneys, muscles, and heart.	USA
Tea tablets by Nanolabs™ Global	Hemp oil contains polyphenols (primarily flavonoids, stilbenes, and lignan-amides), alkaloids, cannabinoids, and terpenoids.	Burns fat, hydrates, and boosts metabolism.	USA

**Table 2:** Enhancing the quality and bioavailability of some food products through the use of nanoencapsulation for mineral fortification.

Type of Food	Mineral Type: Form	Detrimental Effects and Bioavailability	References
Petit Suisse cheese	Fe: C_12_H_24_FeO_14_	The industrial-enriched sample exhibited a significantly increased rate of Fe bioavailability.	[60]
Cheddar cheese	Zn: ZnSO_4_	The cheese fortified with zinc, containing five times more zinc than the control sample, had a harder texture. However, there were no differences seen in terms of its sensory qualities and overall quality.	[61]
Bread	Zn: H_14_O_11_SZn	All groups experienced a notable rise in serum Zn and Fe levels, except for the control group. The group that consumed high-Zn bread showed considerably higher Zn and Fe absorption compared to the group that consumed low-Zn bread.	[62]
Oat biscuit	The product is a premix including 11 minerals and 13 vitamins.	Whey protein fortification imparts a flavor that makes it less popular compared to commercial alternatives.	[63]

## Data Availability

All data are available upon request.
